# Elderly hospitalization and the New-type Rural Cooperative Medical Scheme (NCMS) in China: multi-stage cross-sectional surveys of Jiangxi province

**DOI:** 10.1186/s12913-016-1638-5

**Published:** 2016-08-24

**Authors:** Bingbing Pan, Zhaokang Yuan, Jiaojiao Zou, Daniel M. Cook, Wei Yang

**Affiliations:** 1 School of Public Health, Nanchang University, Nanchang, People’s Republic of China; 2 School of Community Health Sciences, University of Nevada, Reno, USA

**Keywords:** Elderly, Utilization of health services, New-type rural cooperative medical scheme (NCMS), Rural health, China

## Abstract

**Background:**

Studies assessing the impacts of China’s New-type Rural Cooperative Medical Scheme (NCMS) reform of 2003 among rural elderly have been limited.

**Method:**

Multistage stratified cluster sampling household surveys of 1838, 1924, 1879, 1888, 1890 and 1896 households from 27 villages in Jiangxi province were conducted in 2003/2004, 2006, 2008, 2010, 2012 and 2014. Data from older adults age 65 and above were analyzed. Weighted logistic regression was applied to find factors of elderly hospitalization services.

**Results:**

Since 2003, hospitalization rates for elderly increased, while rates of patients leaving against medical advice and patients avoiding the hospital decreased (*P* < 0.05). Factors associated with a higher likelihood of reporting hospitalization in the past year for elderly were the per-capita financial level V in 2012 for NCMS (Adjusted Odds Ratios [aOR]: 2.295), the level VI in 2014 (aOR: 3.045) versus the level I in 2003 and chronic disease (aOR: 2.089) versus not having a chronic disease. Lower rate of elderly left against medical advice was associated with the financial level V in 2012 (aOR: 0.099) versus the level I. The higher rate of hospital avoidance was associated with chronic disease status (aOR: 5.759) versus not having a chronic disease, while the lower rate was associated with the financial level VI in 2014 (aOR: 0.143) versus the level I. Among reporting reasons for elderly hospital avoidance, the cost-related reasons just dropped slightly over the years.

**Conclusions:**

NCMS improved access to health services for older adults. The utilization of hospitalization services for rural elderly increased gradually, but cost-related barriers remained the primary reporting barrier to accessing hospitalization services.

**Electronic supplementary material:**

The online version of this article (doi:10.1186/s12913-016-1638-5) contains supplementary material, which is available to authorized users.

## Background

With the developing global economy and the gradual extension of human life expectancy, the aging population has become a significant public health problem worldwide [1]. The world is aging rapidly, nearly 60 countries of which have become ageing countries [2]. According to the standard of age composition from the United Nations, a society is aged if the population of 65 years old and above is more than 7 % of the total population [3]. The China Statistical Bureau reports that 88 million people in China were over 65 years old in the year 2000, accounting for 7.0 % of the total population, meaning that China has become an aging country [4]. Many researchers have worked on the demands and utilization of health services [5], the equity of health services [3], and Fall risk-increasing drugs and falls [6] by the elderly. Older adults are more likely to suffer from disease and need hospitalization more urgently [5, 7], resulting in a larger increase of both direct and indirect medical care costs [3]. Most nations have established medical insurance schemes to reduce high medical expenses for individuals. Government health care finance policy has affected the utilization of health services in the United States [8], Korea [9], Vietnam [10], Singapore [11] and elsewhere.

China has struggled to establish a successful medical insurance scheme for several years. Based on the People’s Commune system, the traditional Cooperative medical scheme (CMS) of China was thriving in the 1950s but after a recession with the people’s commune system collapse in 1980s, less than 5 % of farmers were covered by health insurance in the late 1990s [12]. Soon, the burden of disease was increasing poverty across rural China, and over 30 % of the rural population who were not presenting for hospitalization despite physician orders due to economical difficulty in 2003 [13]. Meanwhile, the aging population in China has continued to increase, and about 70 % of elderly population who need specialized health services live in rural areas [3]. In response to those situations, the Chinese government implemented the New-type rural cooperative medical scheme (NCMS) in 2003, which provided increasing funding for hospitalization-based medical services among people living in rural area. NCMS is funded by individuals, collectives and government together, and is organized, guided and supported by government. Farmers are allowed to participate voluntarily. The major goal of NCMS is to solve the problem of poverty caused by diseases which require hospitalization services.

Jiangxi province provides a typical example of the NCMS implementation. In Jiangxi province: 1) the per-capita financing level of NCMS had increased gradually from 30 Yuan in the first years, including cost sharing (consisting of 20 Yuan per-person per-year from government and 10 Yuan each person from individuals), to 370 Yuan in 2014 (320 Yuan from government and 70 Yuan from individuals); 2) risk pooling is based on the county level. The hospitalization reimbursement proportion increased each year as well. In 2003, the reimbursement rate for hospitalization in Jiangxi province at designated medical institutions varied by hospital levels, with townships at 60 %, counties at 40 %, higher level than county at 30 % and non-designated hospitals at 0 %, in 2003, increasing to 90, 80, 50, and 35 %, respectively in 2014. 3) The coverage of NCMS has been over 90 % since 2008 [14].

Several studies have reported evidenced that patients who had participated in NCMS were more likely to look for health care services [15–18]. Zou [19] demonstrated that NCMS improved the utilization of hospitalization services for rural residents. Yu [20] reported that outpatient service utilization has not significantly changed under NCMS, while utilization of hospitalization service has increased. Babiarz [21] showed an increase of 5 % in village clinic use was associated with participation in NCMS, but no change in overall medical care use. As well, out of pocket medical cost went down by 19 % and exposure to financial risk fell by 24–63 %. In contrast, one study demonstrated that the effects of NCMS are limited [22]. Early studies reported primary analysis of the impacts of NCMS on the utilization of hospitalization service in population of all ages, but little has been known about the impacts on elderly people. Nevertheless, the elderly who are in urgent demand of health service and have no economical sources should be far more affected by NCMS. In addition, some studies were out-of-date in design or lacked vertical comparisons.

Hence, this article specifically focused on elderly hospitalization from baseline (2003/2004) before the NCMS implemented, to 2014 when its coverage expanded to almost all in rural areas of Jiangxi province, China. Hospitalization was selected to measure the utilization of medical service, for the poor elderly should be far more subjected to the high hospitalization expense; three indexes, including 1) the rate of hospitalization, 2) left against medical advice and 3) hospital avoidance for elderly were used as proxy to measure hospitalization services utilization in our study [19]. Weighted data intended to make the sample, which was derived from complex sampling methods [23], better represent the population of elderly in Jiangxi province. This article is to attract more attention to the rural elderly and to evaluate the implementation effects of NCMS in Jiangxi province, providing a policy basis for improving the Health Care System Reform.

## Methods

### Data collection

These materials were collected from six household surveys conducted at base-line (2003/2004) before the NCMS implemented and the five follow-up surveys (2006, 2008, 2010, 2012, and 2014) after the NCMS implementation. All counties (70) in Jiangxi province were divided into three tiers according to the average income sorted low from high. County of Xiushui among the low-income level tier, county of Wuyuan among middle income-level tier, and county of Luxi among high income-level tier were selected from pilot counties chosen by government. Using a multistage stratified random cluster sampling method, three townships were randomly selected from each sample county (total of nine townships), three administrative villages were randomly selected from each sample township (total of 27 administrative villages). At last we had investigated approximately all of households in each sample administrative village.

The questionnaires were completed by asking the head of the household about the information from each family member (another family member who is over 18 years old will be interviewed if the head of the household isn’t at home). The questionnaire, including three part (part of general information for the farmer family, part of farmer family health, part of the utilization of medical services, medical costs, and reimbursement fees), was recommended by the Ministry of Health of China and adjusted slightly in the light of the actual situation and the purpose of the study (Additional files 1 and 2). Data on older adults (aged 65 and above) were separated from aggregate data. We had 100 % response rates in all surveys, probably due to the support of local governments. To ensure comparability, we had followed up the same villages in six surveys.

### Data weighting method [23–25]

The related data of participants were weighted to ensure the sample, which come from complex sampling, better represent the population of elderly in Jiangxi province by reduced bias.

The base weight W_base_ = W_1_ × W_2|1_ × W_3|2,1_. W_base_ is the base sampling weight of individual, W_1_ is sampling weight of stage one, W_2|1_ is the sampling weight of stage two, W_3| 2,1_ is the sampling weight of stage three. W_i_ is the sampling weight of individual and is equal to the reciprocal of individual sampling probability Pi, meaning W_i_ = 1/P.

The adjust weight: ${W}_{adj\_ij}={N}_{ij}/{\displaystyle \sum_{m=1}^{n_{ij}}{W}_{ijm},\kern0.24em {w}_{adj-ij}}$ is the adjusted weight for individual, *N*_*ij*_ refers to the individual ensemble of certain category after cross classification of important auxiliary variables such as gender (i = 1, 2) and age (j = 1, 2, 3), ${\displaystyle \sum_{m=1}^{n_{ij}}{W}_{ijm}}$ is the base weight sum of certain group after cross classification.

The final weight of individual $W={W}_{base}\times Wadj\_ij={\mathrm{W}}_1\times {\mathrm{W}}_{\left.2\right|1}\times {\mathrm{W}}_{3\left|2,1\right.}\times {N}_{ij}/{\displaystyle \sum_{m=1}^{n_{ij}}{W}_{ijm}}$

### Sample size estimate

The sample size is estimated by the formula [26]

$$ \mathrm{n}=\frac{{z^2}_{\alpha /2}\times p\left(1-p\right)}{\delta^2} $$

Where n is sample size and p is the hospitalization rate for base-line survey (*p* ≈ 5 %); Z_α/2_ is normal deviate for two-tailed alternative hypothesis at a level of significance, α is set up as 0.05, So Z_0.05/2_ = 1.96; δ is the desired level of margin of error (usually 0.01). So we manually calculated out *n* = 1825. Owing to our research having used cluster sampling method as well, the sampling error is larger than simple random sampling, so it’s better to investigate more by plus 0.5n [27], finally we got a minimal sample size for cluster sampling as n_1_, thus n_1_ = n + 0.5n = 2737.5 ≈ 2738, which is far below the actual sample size (7500~) for six years (Table 1).

**Table 1:** Description of the sample characteristics after data weighted from 2003/2004 to 2014 [percent, estimator (95 %.CI.)]

Variable	2003/2004	2006	2008	2010	2012	2014
Overall sample						
Total responses (n) ^a^	7792	8080	9586	7506	7857	7810
Total responses (n)	850519	1049234	1110086	1127340	1126276	1464991
Household (n) ^a^	1838	1924	1879	1888	1890	1896
Household (n)	166452	219186	202718	253229	239076	307098
Per-capita financial level for NCMS ^a c^	43	66	123	173	306	370
Per-capita hospitalization cost ^c^	3931.15 (1755.74, 6106.57)	4368.88 (2411.44, 6326.27)	5182.37 (3601.72, 6763.03)	4256.32 (3201.31, 5311.32)	6072.76 (5243.59, 6901.92)	7245.03 (6580.56, 7909.52)
Sex						
Male	52.17 (49.51, 54.82)	52.39 (49.92, 54.85)	51.88 (50.99, 52.77)	51.83 (50.91, 52.75)	51.74 (50.85, 52.63)	51.78 (50.79, 52.78)
Female	47.83 (45.18, 50.49)	47.61 (45.15, 50.08)	48.12 (47.23, 49.01)	48.17 (47.25, 49.09)	48.26 (47.37, 49.15)	48.22 (47.22, 49.21)
Age						
0 ~ 64	93.38 (90.62, 95.37)	93.44 (90.94, 95.29)	92.80 (89.94, 94.89)	92.80 (90.56, 94.55)	92.75 (91.99, 93.45)	91.54 (89.85, 92.97)
65~	6.62 (4.63, 9.38)	6.56 (4.71, 9.06)	7.20 (5.11, 10.05)	7.20 (5.45, 9.44)	7.25 (6.55, 8.01)	8.46 (7.03,10.15)
Sample of elderly ^b^						
Response (n) ^a^	631	631	833	615	667	766
Sex						
Male	50.48 (47.50, 53.46)	50.48 (48.28, 52.67)	50.36 (46.28, 54.44)	50.05 (45.28, 54.81)	49.74 (45.95, 53.52)	51.30 (46.17, 56.41)
Female	49.52 (46.54, 52.50)	49.52 (47.33, 51.72)	49.64 (45.56, 53.72)	49.95 (45.19, 54.72)	50.26 (46.48, 54.05)	48.70 (43.59, 53.83)
Age						
65~	60.60 (57.17, 63.93)	62.72 (56.88, 68.20)	65.97 (59.09, 72.24)	67.24 (59.52, 74.12)	68.03 (55.38, 78.48)	63.10 (55.05, 70.49)
75~	34.77 (28.53, 41.57)	33.35 (28.16, 38.98)	29.30 (23.85, 35.41)	27.90 (21.00, 36.03)	27.13 (17.68, 39.22)	29.68 (23.48, 36.74)
85~	4.63 (1.85, 11.15)	3.93 (2.87, 5.37)	4.73 (2.81, 7.85)	4.86 (3.17, 7.39)	4.85 (3.21, 7.26)	7.21 (4.96, 10.38)
Occupation						
Farmer	97.30 (93.95, 98.82)	96.57 (94.13, 98.02)	95.53 (92.81, 97.25)	92.65 (81.16, 94.89)	91.25 (84.51, 95.23)	90.09 (87.26, 92.34)
No-farmer	2.70 (1.18, 6.05)	3.43 (1.98, 5.87)	4.47 (2.75, 7.19)	7.35 (3.11, 16.84)	8.75 (4.77, 15.49)	9.91 (7.66, 12.74)
Educational level						
Illiterate	49.80 (43.80, 55.80)	36.78 (30.15, 43.95)	31.03 (23.15, 40.18)	27.23 (20.29, 35.49)	16.43 (11.81, 22.40)	28.74 (24.46, 33.44)
Elementary school	42.82 (36.97, 48.87)	54.20 (46.14, 62.06)	56.17 (46.64, 65.27)	54.87 (44.49, 64.84)	67.91 (58.81, 75.82)	54.00 (37.70, 69.49)
Middle school and above	7.39 (3.68, 14.29)	9.02 (3.99, 19.12)	12.80 (6.41, 23.93)	17.90 (12.30, 25.31)	15.67 (9.08, 25.69)	17.25 (7.67, 34.34)
Marital status						
Married	64.16 (54.25, 72.99)	54.49 (50.50, 58.42)	62.93 (51.03, 73.44)	59.61 (52.70, 66.15)	62.21 (58.77, 65.54)	64.16 (57.08, 70.68)
Unmarried/divorced/widowed	35.84 (27.01, 45.74)	45.51 (41.58, 49.50)	37.07 (26.56, 48.97)	40.39 (33.85, 47.30)	37.79 (34.46, 41.23)	35.84 (29.32, 42.92)
Labor force						
Yes	11.08 (8.27, 14.69)	31.46 (27.23, 36.03)	14.89 (12.03, 18.28)	33.21 (27.59, 39.35)	40.19 (35.34, 45.24)	44.92 (38.38, 51.65)
No	88.92 (85.31, 91.73)	68.54 (63.97, 72.77)	85.11 (81.72, 87.97)	66.79 (60.65, 72.41)	59.81 (54.76, 64.66)	55.08 (48.35, 61.62)
Chronic disease						
Yes	25.87 (21.43, 30.87)	23.10 (15.37, 33.20)	25.69 (17.22, 36.50)	31.43 (27.68, 35.45)	22.70 (19.62, 26.11)	29.62 (24.53, 35.27)
No	74.13 (69.13, 78.57)	76.90 (66.80, 84.63)	74.31 (63.50, 82.78)	64.55 (59.25, 72.32)	77.30 (73.89, 80.38)	70.38 (64.73, 75.47)
NCMS						
Yes	0.00 (0.00, 0.00)	96.66 (89.85, 98.96)	99.29 (97.91, 99.76)	99.63 (97.13, 100.00)	100.00 (100.00, 100.00)	100.00 (100.00, 100.00)
No	100.00 (100.00,100.00)	3.34 (1.04, 10.15)	0.71 (0.24, 2.09)	0.36 (0.05, 2.87)	0.00 (0.00, 0.00)	0.00 (0.00, 0.00)
Per-capita income per year [mean, estimator (95 %.CI.)]^c^	3882.56 (3031.80, 4733.33)	8589.0 (7055.90 10122.21)	6339.38 (4278.85, 8399.91)	8443.24 (7860.20, 9026.29)	8375.49 (7070.40, 9680.57)	15653.19 (6814.34, 24492.04)

^a^ Without weighted the data

^b^ Elderly: aged 65 and above

^c^ Per-capita financial level, Per-capita hospitalization cost and Annual per capita household income were inflated to the year of 2014 using rural consumer price index; their units are RMB (Yuan)

### Indexes construction

The hospitalization rate of elderly means the rate of the elderly in hospitalization as a result of disease compared to the total number of elderly respondents in the past year (%). Hospitalization was measured by asking whether respondents received any hospitalization services in the past year.

The left against medical advice rate of elderly refers to the rate of the number of elderly who left the hospital against medical advice to the number of elderly hospitalization in the past year (%). Left against medical advice was measured by asking whether there was a time in the past year when a doctor advised the respondent to stay in the hospital, but he or she left nonetheless.

The rate of elderly hospital avoidance means the rate of the number of elderly people who should receive hospitalization services but did not, compared to the number of those who should be hospitalized in the past year (%). Hospital avoidance was measured by asking whether there was a time in the past year when a doctor thought the respondent should receive hospitalization services but the patient was never admitted.

If a patient had more than one episode of hospitalization or left against medical advice, or chose hospital avoidance, she/he was only counted once in calculating the three indexes.

### The quality and representativeness of the data

We used Myer’s index to test the data quality, using age as the basis of the logic judgment process [28]. Myer’s index should not be bigger than 60; otherwise the quality of the data was poor for us to analyze [29]. We used goodness for fit test to observe the fitting degree of the distribution of sample data and the distribution of theoretical frequency. If the test results showing no statistical significance (*P* values > 0.05), the representativeness of the data cannot be regarded as poor.

The results of the Myer’s index of this study from 2003/2004 to 2014 were 4.00, 5.12, 12.02, 8.52, 3.54, and 7.07, respectively. Correspondingly, the value of χ^2^ of the test of goodness for fit of sex were 0.0014, 0.0002, 0.0005, 0.1103, 0.0021, 0.5396, *P* values > 0.05. Therefore, the quality and representativeness of the data cannot be regarded as poor.

### Data analysis

The sample data were weighted to be representative of our target population using methods consistent with previous research [24, 25, 30–32]. Myer’s index and goodness for fit test were employed to test the data quality and representativeness [28, 29]. Pearson Chi-Square test was used to compare the differences between groups. The significance level α was set at 0.05. The rates of elderly hospitalization, left against medical advice and hospital avoidance were used to descript the utilization of hospitalization service. We applied weighted logistic regression to find factors of the utilization of hospitalization service. Crude Odds ratio (cOR) was conducted by bivariate logistic regression and adjusted Odds ratio (aOR) was conducted by multivariate logistic regression with stepwise technique. The inclusion criteria of stepwise in the logistic regressions model was set at ≤ 0.05 [33]. Independent variables included per-capita financially level for NCMS (referent group = level I), sex (referent group = female), occupation (referent group = farmer), educational level (referent group = illiterate), marital status (referent group = married), labor force (referent group = non-labor force), chronic disease (referent group = non-chronic disease), per-capita income per year (referent group = group I). The financially level every year for NCMS was inflated into the year of 2014 using rural consumer price index [4] and used as the proxy of effects of NCMS, for the coverage rate of NCMS for elderly has been over 95 % since 2006 (see Table 1). The per capita income per year was inflated into the year of 2014 using rural consumer price index [4] and divided into five groups when sorted from low to high (group I, group II, group III, group IV, group V), for analyzed the effects of per capita income on elderly hospitalization. Microsoft excel 2013 software was used to set up data base, weight the data and draft the figures, PASW Statistics 18.0 software to analyze the data.

## Results

### Sample characteristics

The numbers of elderly/overall sample for the six surveys before data weighted were 631/7792, 631/8080, 833/9586, 615/7506, 667/7857 and 766/7810, respectively. The proportions of elderly number to the number of overall sample of the six surveys after data weighted were 6.62, 6.56, 7.20, 7.20, 7.25, and 8.46 %, respectively, which evidenced that Jiangxi province has been becoming an aging area. The prevalence of chronic disease in half year for elderly from 25.87 % in 2003/2004 increased slightly to 29.62 % in 2014. After inflating the per capita income for elderly, per-capita financial level for NCMS and per-capita hospitalization cost of 2003/2004, 2006, 2008, 2010 and 2012 to 2014 levels (using the rural consumer price index), the per capita income was 15653.19 Yuan in 2014, whose annual average growth rate was 14.96 %; the annual average growth rate for per-capita financial level was 21.72 % from 2003/2004 to 2014, for per-capita hospitalization cost was 6.30 %. The coverage of NCMS for elderly has been over 95 % since 2006 (see Table 1).

### Changes of hospitalization services utilization for elderly

After weighting the data, the rates of elderly hospitalization rose from 6.67 % in baseline (2003/2004) to 18.70 % in 2014 (6.39 % in 2006, 9.43 % in 2008, 10.50 % in 2010, 13.99 % in 2012), the rates of elderly left against medical advice dropped from 21.59 % in 2006 to 9.25 % in 2014 (8.22 % in 2008, 12.91 % in 2010, 2.49 % in 2012), and the rates of elderly hospital avoidance fell from 46.11 % in baseline to 12.19 % in 2014 (35.84 % in 2006, 35.60 % in 2008, 14.91 % in 2010, 7.88 % in 2012). The *χ*^*2*^ test of the three indexes showed statistical significance (*P* < 0.05). See Table 2.

**Table 2:** Bivariate analysis of the three indexes after data weighted [percent, estimator (95 %.CI.)]

Demographic characteristics	The rate of elderly hospitalization	The rate of elderly leaving against medical advice	The rate of elderly hospital avoidance
Year^a^			
2003/2004	6.67 (4.51, 9.76)	-	46.11 (13.85, 82.00)
2006	6.39 (3.20, 12.36)	21.59 (7.82, 47.19)	35.84 (25.05, 48.29)
2008	9.43 (7.65, 11.57)	8.22 (2.51, 23.79)	35.60 (19.81, 55.30)
2010	10.50 (4.50, 22.59)	12.91 (4.73, 30.70)	14.91 (8.56, 24.69)
2012	13.99 (10.74, 18.03)	2.49 (0.96, 6.30)	7.88 (2.06, 25.79)
2014	18.70 (10.50, 31.09)	9.25 (3.49, 22.30)	12.19 (5.99, 23.23)
P^b^	*p* < 0.001	*p* < 0.001	*p* < 0.001
Sex			
Male	11.93 (9.56, 14.80)	11.28 (6.14, 19.81)	19.14 (15.94, 22.80)
Female	11.98 (10.35, 13.83)	7.19 (2.20, 21.05)	21.97 (18.02, 26.49)
p^b^	p > 0.050	*p* < 0.001	*p* < 0.001
Age			
65~	11.39 (8.39, 15.28)	11.51 (6.55, 19.45)	18.78 (11.92, 28.32)
75~	13.84 (10.50, 18.03)	4.36 (1.78, 10.30)	22.00 (15.57, 30.15)
85~	8.24 (5.21, 12.80)	15.11 (5.72, 34.32)	33.76 (13.38, 62.72)
P^b^	*p* < 0.001	*p* < 0.001	*p* < 0.001
Occupation			
Farmer	13.39 (5.67, 28.45)	9.58 (5.72, 15.62)	21.64 (17.57, 26.35)
No-farmer	11.86 (10.19, 13.76)	5.47 (0.95, 25.92)	5.25 (0.62, 32.87)
P^b^	p > 0.050	*p* < 0.001	*p* < 0.001
Educational level			
Illiterate	10.93 (9.56, 12.46)	9.41 (3.22, 24.47)	24.39 (17.73, 32.56)
Elementary school	12.22 (9.49, 15.61)	7.72 (3.59, 15.83)	18.42 (13.45, 24.71)
Middle school and above	15.47 (10.09, 16.92)	14.38 (4.58, 37.01)	22.77 (9.35, 40.00)
P^b^	*p* < 0.001	*p* < 0.001	*p* < 0.001
Marital status			
Unmarried/divorced/widowed	9.73 (7.13, 13.13)	7.75 (3.22, 17.53)	30.84 (18.58, 46.56)
Married	13.35 (11.66, 15.25)	9.92 (5.39, 17.58)	14.79 (6.99, 28.62)
P^b^	*p* < 0.001	*p* < 0.05	*p* < 0.001
Labor force			
Yes	11.20 (9.82, 12.74)	5.42 (2.36, 12.00)	19.80 (9.27, 37.38)
No	13.61 (10.39, 17.63)	11.04 (6.17, 18.98)	21.00 (16.21, 26.76)
P^b^	*p* < 0.001	*p* < 0.001	*p* < 0.001
Chronic disease			
Yes	18.16 (12.73, 25.25)	13.84 (6.23, 27.94)	32.63 (24.66, 41.75)
No	9.69 (8.51, 11.01)	6.09 (2.52, 13.98)	10.31 (6.75, 15.44)
P^b^	*p* < 0.001	*p* < 0.001	*p* < 0.001
Per-capita income per year^c^			
Group I	10.21 (7.75, 13.36)	2.71 (0.36, 17.73)	29.72 (22.43, 38.22)
Group II	9.84 (7.76, 12.41)	23.99 (10.42, 46.14)	30.04 (16.61, 48.07)
Group III	10.75 (8.13, 14.08)	4.80 (1.01, 20.01)	15.33 (8.34, 26.47)
Group IV	13.44 (10.64, 16.85)	7.88 (2.83, 20.11)	20.23 (14.43, 27.60)
Group V	14.60 (7.53, 26.41)	8.36 (2.36, 25.65)	12.01 (7.75, 18.13)
P^b^	*p* < 0.001	*p* < 0.001	*p* < 0.001

^a^ The survey of leaving against medical advice started in 2006

^b^ Pearson Chi-Square test

^c^ The per capita income per year was inflated to the year of 2014 using rural consumer price index and divided into five groups when sorted from low to high (V is the highest)

### Bivariate analysis for the utilization of elderly hospitalization services

The hospitalization rates of the elderly with different demographic characteristics including age, educational level, marital status and labor force, chronic disease, per-capita income per-year demonstrate statistical significance (*P* values <0.05). The rates of elderly who left against medical advice and who avoided hospital with different demographic characteristics including sex, age, occupation, educational level, marital status and labor force, chronic disease, per-capita income per-year showed statistical significance (all *P* values <0.05). See Table 2.

### Weighted logistic regression analysis for influences factors of the utilization of elderly hospitalization services

To assign variables for the weighted logistic regression model, per-capita financial level, sex, age, occupation, educational level, marital status, labor force, and chronic disease, per capita income per year were took as independent variable, and the dependent variables were the rates of elderly hospitalization, left against medical advice and hospital avoidance. See Appendix.

Logistic regression analysis after data weighted PASW Statistics 18.0 software was employed to carry out logistic regression of complex sample. Crude Odds ratio (cOR) was conducted by bivariate logistic regression and adjusted Odds ratio (aOR) was conducted by multivariate logistic regression with with stepwise technique. In Table 3, independent variables of equation for the three indexed were displayed below.

**Table 3:** Independent variables in the equation of the three indexes and the estimation value of OR

Variable	Elderly hospitalization		Elderly leaving against medical advice		Elderly hospital avoidance	
	cOR (95 %.*CI*)	aOR (95 %.*CI*)	cOR (95 %.*CI*)	aOR (95 %.*CI*)	cOR (95 %.*CI*)	aOR (95 %.*CI*)
Per-capita financial level for NCMS ^a^						
Level II (2006)	0.955 (0.384, 2.315)	0.967 (0.359, 2.610)	-	-	0.653 (0.089, 4.783)	0.648 (0.052,8.076)
Level III (2008)	1.457 (0.956, 2.222)	1.456 (0.847, 2.504)	0.325 (0.080, 1.316)	0.229 (0.033, 1.607)	0.646 (0.059, 7.105)	0.495 (0.021, 11.912)
Level IV (2010)	1.641 (0.844, 3.190	1.550 (0.754, 3.185)	0.538 (0.091, 3.194)	0.413 (0.144, 1.185)	0.205 (0.039, 1.081)	0.205 (0.020, 2.139)
Level V (2012)	2.276 (1.452, 3.567) ^**^	2.295 (1.401, 3.759) ^**^	0.093 (0.022, 0.389) ^**^	0.099 (0.033, 0.293) ^**^	0.100 (0.006, 1.712)	0.073 (0.003, 1.984)
Level VI (2014)	3.219 (1.187, 8.732) ^*^	3.045 (1.183, 7.834) ^*^	0.370 (0.115, 1.188)	0.407 (0.102, 1.618)	0.162 (0.047, 0.556) ^*^	0.143 (0.024, 0.875) ^*^
Sex						
Male	0.995 (0.795, 1.246)	0.900 (0.695, 1.165)	0.610 (0.134, 2.772)	1.762 (0.486, 6.391)	0.841 (0.656, 1.078)	1.269 (0.876, 1.837)
Age						
75~	1.250 (0.685, 2.282)	1.438 (0.690, 2.996)	2.853 (1.265, 6.434) ^*^	0.390 (0.142, 1.070)	1.220 (0.498, 2.986)	1.295 (0.610, 2.751)
85~	0.699 (0.501, 0.975) ^*^	0.802 (0.396, 1.621)	0.731 (0.201, 2.662)	1.422 (0.280, 7.224)	2.205 (0.465, 10.475)	2.555 (0.720, 9.072)
Occupation						
No-farmer	1.149 (0.445, 2.967)	0.979 (0.391, 2.450)	0.546 (0.123, 2.429)	0.743 (0.052, 10.696)	0.201 (0.019, 2.169)	0.208 (0.015, 1.872)
Educational level						
Elementary school	1.135 (0.829, 1.555)	1.019 (0.849, 1.222)	0.806 (0.444, 1.463)	1.357 (0.421, 4.372)	0.700 (0.424, 1.155)	1.300 (0.809, 2.092)
Middle school and above	1.232 (0.858, 1.769)	0.990 (0.716, 1.368)	1.617 (0.209, 12.487)	1.684 (0.545, 5.204)	0.813 (0.285, 2.318)	1.620 (0.735, 3.574)
Marital status						
Unmarried/divorced/widowed	0.699 (0.509, 0.961) ^*^	0.662 (0.419, 1.044)	0.763 (0.267, 2.182)	0.832 (0.218, 3.181)	2.568 (0.603, 10.940)	2.946 (0.762, 11.383)
Labor force (yes)	1.249 (0.962, 1.622)	1.051 (0.697, 1.584)	0.462 (0.166, 1.285)	0.388 (0.110, 1.375)	0.929 (0.295, 2.919)	0.876 (0.405, 1.895)
Chronic disease (yes)	2.069 (1.355, 3.159) ^**^	2.089 (1.450, 3.011) ^**^	2.477 (0.607, 10.109)	2.433 (0.542, 10.931)	4.213 (2.007, 8.842) ^**^	5.759 (2.943, 11.268) ^**^
Per-capita income per year ^b^						
Group II	0.960 (0.654, 1.407)	1.155 (0.638, 2.091)	1.349 (0.300, 9.109)	2.516 (0.805, 7.862)	1.016 (0.454, 2.273)	1.187 (0.358, 3.940)
Group III	1.059 (0.724, 1.548)	1.089 (0.796, 1.489)	1.812 (0.158, 20.834)	1.920 (0.352, 10.485)	0.428 (0.176, 1.040)	0.762 (0.213, 2.725)
Group IV	1.365 (0.889, 2.098)	1.033 (0. 695, 1.536)	3.077 (0. 637, 14.869)	6.887 (2.035, 23.303)^*^	0.600 (0.354, 1.016)	0.932 (0.235, 3.694)
Group V	1.502 (0.709, 3.186)	1.103 (0.640, 1.900)	3.282 (0.273, 39.525)	2.206 (0.219, 22.238)	0.323 (0.147, 0.708)^*^	0.464 (0.115, 1.877)

^a^ The survey of leaving against medical advice started in 2006

^b^ The per capita income per year was inflated to the year of 2014 using rural consumer price index and divided into five groups when sorted from low to high (V is the highest)

^**^ *p* < 0.01, ^*^ *p* < 0.05

Factors were associated with a higher likelihood of reporting hospitalization in the past year for elderly were the per-capita financial level V in 2012 (aOR: 2.295), the level VI in 2014 (aOR: 3.045) versus the level I in 2003 and chronic disease (aOR: 2.089) versus not having a chronic disease. Lower rate of elderly left against medical advice was associated with the financial level V in 2012 (aOR: 0.099) versus the level I. Higher rate of hospital avoidance was associated with chronic disease status (aOR: 5.759) versus not having a chronic disease, while the lower rate was associated with the financial level VI in 2014 (aOR: 0.143) versus the level I. All of the above variables in the models showed statistical significance (*P* values ≤ 0.05).

### The analysis of reporting reasons for elderly left against medical advice and hospital avoidance

**Fig. 1:**
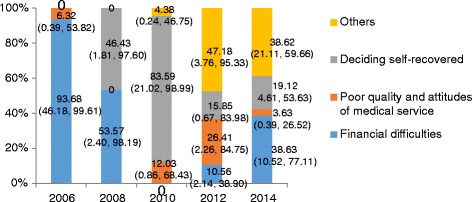
The proportion of reporting reasons for elderly leaving against medical advice

**Fig. 2:**
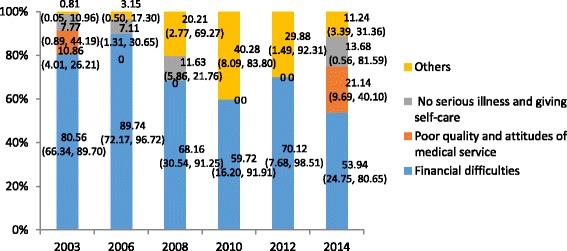
The proportion of reporting reasons for elderly hospital avoidance

Among the reporting reasons for elderly left against medical advice (Fig. 1) (e.g. financial difficulties, poor quality and attitudes of medical service, deciding self-recovered and others), financial difficulties played an important role before 2010, but declined sharply in 2010 and then presented a rebound trend. In the reporting reasons of elderly hospital avoidance (Fig. 2), including financial difficulties, poor quality and attitudes of medical service, no serious illness and giving self-care and others, the proportion of financial difficulties declined slightly. But it still was the main reason in all of the years.

## Discussion

In this study, the rates of elderly hospitalization, patient-initiated early discharge, and hospital avoidance have been increasing, dropping and dropping, respectively from 2003 to 2014. This reveals that the appropriate utilization of elderly hospitalization service has been facilitated in these years. The potential reason may be some health care reforms carried out in China, like NCMS. The policy had a primary aim to resolve economic barriers to hospitalization. The actual proportion of hospitalization reimbursement (the proportion of farmers’ expense for hospitalization reimbursed by relevant departments to all of the expenditures) increased in Jiangxi province, from 40.55 % in 2008 [34] to 55.09 % in 2014 [35]. Nearly half of the hospitalization cost could be rembursed after 2008, which eased the burden of hospitalization services for a large sector of rural residents who participated in NCMS. This means participants would be more likely to be hospitalized when illness warranted, rather than cobbling together other various remedies. This is in agreement with another study that found NCMS promoted the utilization of inpatient service among the elderly [36]. Even though other health care reforms, such as public hospital reform and essential medicine reform, were carried out as well in recent years, the new systems are not without limits. Public hospital reform was brought to life among county-level hospitals in 2013 and covered 13.13 % of overall counties in Jiangxi province [37]. The coverage rate of public hospital reform in Jiangxi was only 22.22 % in 2014 [35]. Essential medicine reform was brought to life by the government of China in 2009 [38] and covered only the primary health care institutions (township health center, village clinic) in 2014, while the availability of hospitalization services for rural residents was overwhelmingly county-level hospitals [35]. In addition, an empirical study demonstrated that, compared to non-pilot medical institutions, the total hospitalization cost in the pilot primary health care institutions where the essential medicine reform as implemented did not decrease, but rather increased by 15 % [39]. Furthermore, in the present study, we found public hospital reform and essential medicine reform had not reduced hospitalization costs as well, because the annual average growth rate for per-capita hospitalization cost from 2003 to 2014 was 6.30 %. Above all, the effects of the two reforms on elderly hospitalization were limited.

In this study, we found that the elderly participants in 2012 (with a financial level of V, aOR = 2.295) and in 2014 (with a financial level of VI, aOR = 3.045) are more likely to be hospitalized, compared to ones in 2003 (financial level I); the rates of elderly left against medical advice was decreased by the financial levelV in 2012 (aOR = 0.099); the elderly participants in 2014 (with a financial level of VI, aOR = 0.143) are less likely to avoid being hospitalized. In the logistic regression models of hospitalization services for elderly, the financial level was taken as the proxy of NCMS, since the coverage of NCMS for elderly has been over 95 % since 2006 in this study, and the number of non-NCMS group is too small for accurate comparison. Because the financing level of NCMS had been increasing substantially and its annual average growth rate was 21.72 % from 2003 to 2014 in Jiangxi, this would be an effective measurement for NCMS. These data indicated that the effects of NCMS policy on hospitalization services were evident in the most recent few years but limited in the first years, probably due to the lower financial level and reimbursement rate in these years. Although respondent income levels have also increased a lot in this decade, the variable of income level was not associated with the utilization of hospitalization services in the multivariate logistic regression analysis.

Meanwhile, the utilization of hospitalization service for elderly was also associated with the prevalence of chronic diseases (aOR = 2.089). As the population ages, the system receives additional patients who are older adults with chronic diseases, which leads to more hospitalizations.

In addition, we found that the elderly suffering from chronic diseases were more likely to avoid being hospitalized even though their doctor advised treatment (aOR = 5.759). This is consistent with the study conducted in Chongqing [40], which observed that the elderly with chronic diseases affected the hospitalization utilization. The potential reason is that the elderly, owing to expensive medical cost and fewer economic resources, have no money to be hospitalized. The concordant result was found in this study: financial difficulty still was the primary reported reason for elderly hospital avoidance in all of the years examined. In addition, the proportion of Poor quality and attitudes of medical service among reasons of hospital avoidance in 2014 was relatively higher, potentially in that as people's living level improved these years, the demand for quality and attitudes of medical service might increase. The quality of medical service in the past may be the same as that in the present, or worse, but the people were poor before and the demand for being hospitalized could be not fulfilled, not to mention the demand for quality and attitudes of medical service. Furthermore, the elderly, who may have been suffering from chronic diseases for a longer time, took the disease status for granted and lost trust in the doctors, because of the poor quality of medical services.

Compared with the national level, the hospitalization rate for the elderly was 9.43 %, lower than the whole country for both the urban elderly population (19.36 %) and the rural elderly population (12.94 %) in 2008 [41]. The rate of elderly hospital avoidance was 35.60 %, higher than for the whole country in both the urban elderly population (23.8 %) and the rural elderly population (31.4 %) in 2008 [41]. Although the utilization level of elderly hospital service improved gradually, the effects in rural areas of Jiangxi Province were significantly less than for the national level both in urban and rural areas. The potential explanation is that the funding for NCMS in Jiangxi province is lower than that in the provinces with higher economic scales. Moreover, the financial levels for NCMS in Jiangxi province is far lower than medical insurance for urban residing individuals in China and the reimbursement range is narrower, the type of reimbursable medicine being more limited and the amount of reimbursement being far lower [40] continue to pay more attention to the demand and utilization of elderly health services, especially for the elderly with chronic diseases, and should provide more medical assistance to elderly population with financial difficulties.

### Limitations

Some data in our survey, like gross household income, were collected on the basis of personal recall and as such recall bias may serve as a limitation of the current study. Even so, several global surveys (e.g. the Behavioral Risk Factor Surveillance System in the USA [42]) are also subject to recall bias. Thus, this is a common limitation in survey research. However, the response rates of all surveys were 100 %, maybe indicating non-response bias was not a significant issue. This is due in large part to the village leaders in each village guiding researchers to interview rural residents. At the same time, government support may lead to selection bias, but we had almost investigated all of households in the village, this bias may be not a significant issue.

There are three variable selection procedures in logistic regression, namely forward, backward and stepwise. Each variable selection procedures have limitations. As to forward technique, the introduction of the following variables would make the variables previously entered be insignificance. If using backward technique, the number of variables selected is more than the number using forward technique. However, if there is large number of independent variables or highly correlations between them, it will result in incorrect associations. Stepwise technique overcomes the limitations of the two techniques above, but that not to say it is the best way to select variables.

We were unable to investigate the exact same individuals in every year of the study. Thus, potential migratory flow within China should be taken into account when interpreting the results. However, we did measured changes in the same villages.

Myers index is a way of measuring the serious of investigators filling in questionnaire, namely no phenomenon of the accumulation within age of the tail when investigators filling in variable of age. However, it has no ability to illustrate other quality problems.

## Conclusion

In rural areas of Jiangxi province, with the continued improvement of NCMS policy, the utilization of hospitalization health services for the rural elderly increased gradually, which demonstrated that the implementation of NCMS in Jiangxi province achieved positive effects. But the effects were significantly less than at the national level both in urban and rural areas, and cost-related barriers still were the primary reported reason given for limited access to in-patient services. Furthermore, in the multivariate logistic regression analysis, the factors that influenced elderly hospital admissions were the high level financial level of NCMS, and the prevalence of chronic diseases. Hence, the Chinese government should continue to pay more attention to the demand and utilization of elderly health services, especially for the elderly with chronic diseases, and should provide more medical assistance to elderly population with financial difficulties.

## Appendix

**Table 4:** Variable assignment of the potential factors of elderly hospitalization service

Factors	Variable name	Factor assignment
Dependent variables		
Hospitalization	Y_1_	1 = Yes, 0 = No(referent)
Leaving against medical advice	Y_2_	1 = Yes, 0 = No(referent)
Hospital avoidance	Y_3_	1 = Yes, 0 = No(referent)
Independent variables		
Per-capita financial level for NCMS^a^	X_1_, X_2_,	Level I(43Yuan)(referent): X_1_ = 0, X_2_ = 0, X_3_ = 0, X_4_ = 0, X_5_ = 0
	X_3,_ X_4_, X_5_	Level II(66Yuan,2006): X_1_ = 1, X_2_ = 0, X_3_ = 0, X_4_ = 0, X_5_ = 0
		Level III(123Yuan, 2008): X_1_ = 0, X_2_ = 1, X_3_ = 0, X_4_ = 0, X_5_ = 0
		Level IV(173Yuan, 2010): X_1_ = 0, X_2_ = 0, X_3_ = 1, X_4_ = 0, X_5_ = 0
		Level V(306Yuan, 2012): X_1_ = 0, X_2_ = 0, X_3_ = 0, X_4_ = 1, X_5_ = 0
		Level VI(370Yuan, 2014): X_1_ = 0, X_2_ = 0, X_3_ = 0, X_4_ = 0, X_5_ = 1
Sex	X_6_	1 = Male, 0 = Female(referent)
Age	X_7_, X_8_	65 ~ (referent): X_7_ = 0, X_8_ = 0
		75~: X_7_ = 1, X_8_ = 0
		85~: X_7_ = 0, X_8_ = 1
Occupation	X_9_	1 = No-farmer, 0 = Farmer(referent)
Educational level	X_10_, X_11_	Illiterate(referent): X_10_ = 0, X_11_ = 0
		Elementary school: X_10_ = 1, X_11_ = 0
		Middle school and above: X_10_ = 0, X_11_ = 1
Marital status	X_12_	1 = Unmarried/divorced/widowed, 0 = Married(referent)
Labor force	X_13_	1 = Yes, 0 = No(referent)
Chronic disease	X_14_	1 = Yes, 0 = No(referent)
Per capita income per year^b^	X_15_, X_16_,	Group I(referent): X_15_ = 0, X_16_ = 0, X_17_ = 0, X_18_ = 0
	X_17,_ X_18_	Group II: X_15_ = 1, X_16_ = 0, X_17_ = 0, X_18_ = 0
		Group III: X_15_ = 0, X_16_ = 1, X_17_ = 0, X_18_ = 0
		Group IV: X_15_ = 0, X_16_ = 0, X_17_ = 1, X_18_ = 0
		Group V: X_15_ = 0, X_16_ = 0, X_17_ = 1, X_18_ = 0

^a^ When the index of leaving against medical advice analyzed, the referent was 2006

^b^The per capita income per year was inflated to the year of 2014 using rural consumer price index and divided into five groups when sorted from low to high (V is the highest)

## Additional files

Additional file 1: Questionnaire 1. The questionnaire of farmer family health in Jiangxi Province. (DOCX 21.2 kb) Additional file 2: Questionnaire 2. The questionnaire of medical service utilization and cost. (DOCX 22. kb) Additional file 3: Overall sample. The raw data information about the overall sample, such as sex, age and so on. (DOCX 12 kb)

## Abbreviations

aOR: Adjusted Odds Ratios
CMS: Traditional Cooperative Medical Scheme
cOR: Crude Odds Ratio
NCMS: New-type Rural Cooperative Medical Scheme
