# Supplementary material for: Elderly hospitalization and the New-type Rural Cooperative Medical Scheme (NCMS) in China: multi-stage cross-sectional surveys of Jiangxi province
Source: BMC Health Serv Res. 2016 Aug 24;16(1):436. doi: 10.1186/s12913-016-1638-5 (PMC4997654; doi:10.1186/s12913-016-1638-5)
Supplement: Additional file 1: — Questionnaire 1. The questionnaire of farmer family health in Jiangxi Province. (DOCX 21.2 kb) [file 12913_2016_1638_MOESM1_ESM.docx]

**Table 1 The questionnaire of farmer family health in Jiangxi Province**

County code township code administrative villages code

Householder’s name NO.□□□□□□

**Basic information**

Total family members (in [household register](http://dict.youdao.com/w/household register/" \l "keyfrom=E2Ctranslation)): (persons) Annual household income (last year): (Yuan)

[New](http://dict.youdao.com/w/new/) [Rural](http://dict.youdao.com/w/rural/) [Co-operative](http://dict.youdao.com/w/co-operative/) [Medical](http://dict.youdao.com/w/medical/) Scheme: (①participate/②not)

Paying for the New Rural Co-operative Medical Scheme per person (Yuan)

| 1.Number of family member | 1 | 2 | 3 | 4 | 5 | 6 | 7 | 8 |
| --- | --- | --- | --- | --- | --- | --- | --- | --- |
| 1. Relationship with householder：   ①Householder ②Wife ③ Sons and daughters  ④Grandchildren ⑤Parents ⑥Grandparents ⑦Brothers and  sisters  ⑧Others |  |  |  |  |  |  |  |  |
| 3.Are you labor force of your family ?: ①Yes ②No |  |  |  |  |  |  |  |  |
| 4.Sex：①Male ②Female |  |  |  |  |  |  |  |  |
| 5.Age(years old)： |  |  |  |  |  |  |  |  |
| 1. [Marital](http://dict.youdao.com/w/marital/) [status](http://dict.youdao.com/w/status/)：   ①Unmarried ②In marriage ③Divorce ④Widowed ⑤Other |  |  |  |  |  |  |  |  |
| 1. Education level：   ①Illiterate ②Elementary ③Middle school  ④High school ⑤[Technical](http://dict.youdao.com/w/technical/) [secondary](http://dict.youdao.com/w/secondary/) [school](http://dict.youdao.com/w/school/)  ⑥Junior college ⑦College and above |  |  |  |  |  |  |  |  |
| 1. Occupational [status](http://dict.youdao.com/w/status/)：①Farmer   ②Worker of township enterprises ③Rural doctor of village ④Village cadres ⑤Teacher  ⑥Business services worker ⑦Student  ⑧Children of preschool  ⑨leave ones hometown and look for a job ⑩Others |  |  |  |  |  |  |  |  |
| 9. Whether or not you were diagnosed of chronic diseases by the doctor in recent half year: ①Yes (turn to table 2) ②No |  |  |  |  |  |  |  |  |
| 10. Whether or not you got inpatient service in recent year: ①Yes (turn to table 2) ②No (turn to number 13) |  |  |  |  |  |  |  |  |
| 11.Was there a situation that you left against medical advice when you were in hospital in recent year: ①Yes ②No (turn to number 13) |  |  |  |  |  |  |  |  |
| 12.The main reason of leaving against medical advice (Single Selection)：① Considering getting well ②Have no time for a hospital stay and deciding self-recovered ③Poor health services ④Regard the hospital is unable to treat this disease ⑤Economic difficulties ⑥other |  |  |  |  |  |  |  |  |
| 13.Was there a situation that the doctor thought you should be in hospital but you did not want to in recent year：①Yes ②No (end) |  |  |  |  |  |  |  |  |
| 1. The main reason of hospital avoidance (Single Selection)：①Self-treatment ②Regard as light illness and it is not necessary to be hospitalized③Have no time ④Economic difficulties ⑤Poor health services ⑥Regard the hospital is unable to treat this disease ⑦Be afraid of hospitalization ⑧No beds ⑨others |  |  |  |  |  |  |  |  |

Investigator: Date:
