# Supplementary material for: Elderly hospitalization and the New-type Rural Cooperative Medical Scheme (NCMS) in China: multi-stage cross-sectional surveys of Jiangxi province
Source: BMC Health Serv Res. 2016 Aug 24;16(1):436. doi: 10.1186/s12913-016-1638-5 (PMC4997654; doi:10.1186/s12913-016-1638-5)
Supplement: Additional file 2: — Questionnaire 2. The questionnaire of medical service utilization and cost. (DOCX 22. kb) [file 12913_2016_1638_MOESM2_ESM.docx]

**Table 2 The questionnaire of medical service utilization and cost**

County code township code administrative villages code

Householder’s name NO.□□□□□□

| Number of family member | | |  |  |  |  |  |  |
| --- | --- | --- | --- | --- | --- | --- | --- | --- |
| Village-level medical institutions | [chronic](http://dict.youdao.com/w/chronic/) [disease](http://dict.youdao.com/w/disease/) (in recent half year)  (Only refers to outpatient) | Name of disease |  |  |  |  |  |  |
|  |  | Times |  |  |  |  |  |  |
|  |  | Actual [reimbursement](http://dict.youdao.com/w/reimbursement/) /total cost | / | / | / | / | / | / |
| Township Health Center | [chronic](http://dict.youdao.com/w/chronic/) [disease](http://dict.youdao.com/w/disease/) (in recent half year)  (Only refers to outpatient) | Name of disease |  |  |  |  |  |  |
|  |  | Times |  |  |  |  |  |  |
|  |  | Actual [reimbursement](http://dict.youdao.com/w/reimbursement/) /total cost | / | / | / | / | / | / |
|  | Inpatient (in recent year) | Name of disease |  |  |  |  |  |  |
|  |  | Times |  |  |  |  |  |  |
|  |  | Actual [reimbursement](http://dict.youdao.com/w/reimbursement/) /total cost | / | / | / | / | / | / |
|  |  | Days |  |  |  |  |  |  |
| County Hospital | [chronic](http://dict.youdao.com/w/chronic/) [disease](http://dict.youdao.com/w/disease/) (in recent half year)  (Only refers to outpatient) | Name of disease |  |  |  |  |  |  |
|  |  | Times |  |  |  |  |  |  |
|  |  | Actual [reimbursement](http://dict.youdao.com/w/reimbursement/) /total cost | / | / | / | / | / | / |
|  | Inpatient (in recent year) | Name of disease |  |  |  |  |  |  |
|  |  | Times |  |  |  |  |  |  |
|  |  | Actual [reimbursement](http://dict.youdao.com/w/reimbursement/) /total cost | / | / | / | / | / | / |
|  |  | Days |  |  |  |  |  |  |
| Over county-level-hospitals | [chronic](http://dict.youdao.com/w/chronic/) [disease](http://dict.youdao.com/w/disease/) (in recent half year)  (Only refers to outpatient) | Name of disease |  |  |  |  |  |  |
|  |  | Times |  |  |  |  |  |  |
|  |  | Actual [reimbursement](http://dict.youdao.com/w/reimbursement/) /total cost | / | / | / | / | / | / |
|  | Inpatient (in recent year) | Name of disease |  |  |  |  |  |  |
|  |  | Times |  |  |  |  |  |  |
|  |  | Actual [reimbursement](http://dict.youdao.com/w/reimbursement/) /total cost | / | / | / | / | / | / |
|  |  | Days |  |  |  |  |  |  |

Investigator date:
