# Supplementary material for: Elderly hospitalization and the New-type Rural Cooperative Medical Scheme (NCMS) in China: multi-stage cross-sectional surveys of Jiangxi province
Source: BMC Health Serv Res. 2016 Aug 24;16(1):436. doi: 10.1186/s12913-016-1638-5 (PMC4997654; doi:10.1186/s12913-016-1638-5)
Supplement: Additional file 3: — Overall sample. The raw data information about the overall sample, such as sex, age and so on. (DOCX 12 kb) [file 12913_2016_1638_MOESM3_ESM.docx]

Sampling plan

| Stage | Label | Strata | Clusters | Weights | Size | Method |
| --- | --- | --- | --- | --- | --- | --- |
| 1 | (None) | county code | township code | The final weight of individual | (Read from township probability) | Equal WOR |
| 2 | (None) |  | village code |  | (Read from village probability | Equal WOR |
| 3 | (None) |  | family code |  | (Read from household probability) | Equal WOR |
